# Effects of Non-directional Mechanical Trauma on Gastrointestinal Tract Injury in Rats

**DOI:** 10.3389/fphys.2021.649554

**Published:** 2021-04-15

**Authors:** Lihong Liu, Lianpu Wen, Chuanzhou Gao, Hua Piao, Hui Zhao, Deqin Yu, Liang Zhu, Shuzhuang Li

**Affiliations:** ^1^Department of Physiology, College of Basic Medical Sciences, Dalian Medical University, Dalian, China; ^2^Central Laboratory, Dalian Medical University, Dalian, China

**Keywords:** gastrointestinal tract, mucosal injury, LPS, D-lactate, mechanical trauma

## Abstract

Mechanical trauma can (MT) cause secondary injury, such as cardiomyocyte apoptosis and cardiac dysfunction has been reported. However, the effects of mechanical trauma on gastrointestinal tract is unclear. This study aims to observe the main location and time of gastrointestinal tract injury caused by non-directional trauma and explain the reason of the increase of LPS in blood caused by mechanical injury. Morphological changes in the stomach, ileum and cecum at different time points after MT were observed in this experiment. The results reveal that the injury to the cecal mucosa in the rats was more obvious than that in the ileum and the stomach. The cecal epithelial cell junction was significantly widened at 20 min after MT, and the plasma LPS and D-lactic acid concentrations increased significantly at the same time point. In addition, some bacterial structures in the widened intercellular space and near the capillary wall of the cecal mucosa were detected at 12 h after MT. This finding suggests that the main reason for the increase in LPS in plasma after MT is cecal mucosal injury. This study is important for the early intervention of the gastrointestinal tract to prevent secondary injury after MT.

## Introduction

Mechanical trauma (MT) often occurs in accidents, such as coal mining accidents, traffic accidents, earthquakes and all kinds of falls. MT is one of the most common causes of death and disability in middle-aged and young people under the age of 45 (Hardaway, 2000). With the rapid development of the economy, the transportation, mining and construction industries are also developing rapidly, and increasing numbers of patients with MT have been reported. The medical first aid system at home and abroad is effective, and the primary injury 24 h after MT has been effectively controlled, but secondary myocardial infarction and heart failure, especially multiple organ failure complicated by severe trauma, are still a major threat to the life and health of the patient (Hardaway, 2000; Hori and Kim, 2019; Sun and Yan, 2019).

For secondary myocardial injury, our previous studies showed that MT could cause an increase in tumor necrosis factor-alpha (TNF-α) and other inflammatory factors in blood. TNF-α is the leading cause of myocardial apoptosis (Li et al., 2007a, b; Bicakci et al., 2019), and it is also the most important factor for cardiac dysfunction at the beginning of the post-traumatic period (Ma et al., 2017). The mass release of TNF-α is derived from the mononuclear macrophages activated by lipopolysaccharide (LPS), which is found on the cell wall of gram-negative bacteria (Tao et al., 2005). The trauma caused by a small animal trauma instrument in this experiment was non-directional mechanical trauma (Li et al., 2007a, b; Bicakci et al., 2019), that is, the outer surface of the small animal did not have an open wound; consequently, LPS is not derived from external gram-negative bacteria. The intestine is the largest storage and endotoxin library in the body and the first organ to be affected during traumatic stress. Therefore, it is presumed that internal infection occurs, that is, the bacteria break through the intestinal mucosal barrier into the blood circulation.

The inner surface of the intestine has four layers. Under normal physiological conditions, the structure of the tight junction barrier is complete, which makes it difficult for toxic intestinal contents and molecules to pass through this structure (Ghareeb et al., 2016). In the Wenchuan earthquake, patients were found with no penetrating injury but with intestinal tissue necrosis and bacterial translocation (Awad et al., 2011).

There is no report about the secondary injury of digestive system caused by non-directional mechanical trauma. It is of great significance for the prevention and treatment of secondary multiple organ failure caused by trauma. In this experiment, we observed gastrointestinal tract injury caused by non-directional mechanical trauma in different regions and at different times to determine the key roles and time points of bacterial translocation and toxin into the blood, to explain the causes of the increase in TNF-α in the blood caused by mechanical trauma and to provide a helpful reference for the early intervention of clinical trauma patients.

## Materials and Methods

### Experimental Grouping Design

We randomly divided healthy adult male SPF Sprague Dawley (SD) rats into 10 groups, and the weight range of the rats was 190–230 g at 6–7 weeks. These groups included the control group and the groups at different time points after MT. The groups at different time points after MT included 10 min after MT, 20 min after MT, 45 min after MT, 1.5 h after MT, 3 h after MT, 6 h after MT, 12 h after MT, 24 h after MT, and 48 h after MT, n = 5–8. Rats in the experiment were first anesthetized with phenobarbital (40 mg/kg). Rats in different experimental groups were exposed to room temperature after MT, all rats were fed under normal conditions, and the rats in groups at different times after MT were used for tissue extraction at the appropriate time points.

### Animals and Models

The SD rats were housed in a room maintained at a constant temperature of 20 ± 2°C and under a appropriate humidity, with fasting for 12 h before the experiment. The animals were anesthetized and sacrificed with phenobarbital and were placed in a Noble-Collip drum (200 turns at a rate of 50 rpm) to induce a non-directional trauma as we previously described (Tao et al., 2005; Li et al., 2007a, b). The non-directional trauma model is a classic research model of systemic mechanical trauma (Tao et al., 2005; Noble RL and Collip JB, 1942). With the rotation of the instrument, the animal will fall 400 times due to the effect of gravity, causing MT of the whole body of the SD rats. The number of falls is based on the published papers, which meet the ethical requirements (Tao et al., 2005; Ma et al., 2017). The control group rats are fixed on the inner wall of the rotating plastic with cardboard, and during the rotation of the MT instrument, the rats only rotate with the instrument without trauma induced by MT. All experimental protocols were approved by the animal studies committees of Dalian Medical University (AEE20051) and all efforts were made to minimize the number of animals used and their suffering.

### Plasma Sample Extraction

Blood samples were collected at 20, 45 min, 1.5, 3, 6, 12, 24, and 48 h after MT. The blood samples were collected from the abdominal aorta before digestive tract tissue samples were collected, and 1% heparin sodium solution was added to the blood sample. Heparin sodium solution accounted for 10% of the total plasma volume. Then, the blood samples were centrifuged at 3,000 rpm for 20 min at 4°C to obtain the plasma. The plasma samples are stored at –80°C until analysis.

### Tissue Samples From the SD Rats

The SD rats were anesthetized and sacrificed to collect intestinal samples after plasma sample extraction. A small incision was made on the tip of the cecum and the base of the cecum in the rats. Physiological saline had been stored at 4°C, and the contents were washed out with physiological saline. Approximately 1 mm^2^ of the cecum tissue sample was quickly removed and soaked in 2.5% glutaraldehyde for transmission electron microscopy. The sampling operation was quickly finished. All the remaining cecal tissue was fixed in 10% formalin solution for hematoxylin and eosin (HE) staining for observation under an optical microscope.

At the end of the ileum near the cecum of the SD rats, an approximately 1 cm long ileum intestinal sample was collected and fixed in 10% formalin solution. Samples of approximately 1 cm^2^ size in the middle part of the greater curvature were obtained from the SD rats and fixed in 10% formalin solution. These samples were used for HE staining and observed under an optical microscope.

### Detection of Lipopolysaccharide in Plasma

The LPS concentration was assayed by ELISAs (Rat LPS ELISA Kit, ShangHai Lengton Bioscience Co., Ltd.). The standard was diluted according to the instruction manual, and the samples were added to 1.5 ml EP tube as each point on the standard concentration gradient curve. A blank well was established in the 96-well ELISA plate and served as the zero value. Then, 50 μl of standard substance was used, and 50 μl of streptomycin-HRP was added to the well with the standard substance; next, 10 μl of sample to the sample wells to be tested, and 40 μl of anti-LPS antibody and 40 μl of streptavidin-HRP were added. We covered the plates with sealing membrane, shook them gently and mixed them well. The samples were incubated at 37°C for 60 min. The washing solution was prepared. The sealing film was carefully removed, the liquid was discarded and the samples were dried. Each well was filled with detergent. The wells were left for 30 s, and then, the samples was discarded The procedure was repeated five times. Color development was as follows: first add color agent A (50 μl) to each hole, then add color agent B (50 μl), shake gently and mix well, and develop the color at 37°C in dark for 10 min. Termination was as follows: add 50 μl of termination solution to each pore to terminate the reaction. Determination was as follows: the blank well was zero, and the absorbance (OD value) of each hole was measured at a 450 nm wavelength. The determination was carried out within 10 min after the addition of termination solution. The linear or polynomial regression equation of the standard curve was calculated according to the concentration of the standard product and the corresponding OD value, and then, the corresponding sample concentration value was calculated with the regression equation according to the OD value of the sample.

### Detection of D-Lactic Acid in Plasma

The D-lactic acid concentration was assayed by ELISAs (Rat D-lactic acid ELISA Kit, ShangHai Lengton Bioscience Co., Ltd.). The procedure was performed as described above; 50 μl of standard substance and 40 μl of streptomycin-HRP were added, along with 10 μl of anti-D-lactate antibody and 10 μl of streptavidin-HRP. According to the concentration of the standard product and the corresponding OD value, the polynomial or four parameter curve regression equation of the standard curve was calculated, and then, the corresponding sample concentration value was calculated with the regression equation according to the OD value of the sample.

### Measuring the Mucosal Permeability in Intestinal Mucosa

The mucosal permeability in intestinal mucosa was assayed by testing fluorescein isothiocyanate-4 kD dextran (FD-4) (Sigma, St. Louis, MO, United States). Following a 12-h fasting period, the rats were administered FD-4 via intragastric infusion (25 mg/kg BW, 5 mg/ml). After 4 h, blood was collected and centrifuged at 10,000 × g for 10 min, and the resulting plasma layer was diluted in an equal volume of distilled water. The FD-4 concentrations in the serum were analyzed with a fluorescence spectrophotometer (Thermo Fisher Scientific, United States) at an excitation wavelength of 490 nm and an emission wavelength of 530 nm. Standard curves were obtained by diluting FD-4 in non-treated serum diluted with distilled water (Li et al., 2018; Zeng et al., 2020).

### Western Blot Analysis

Protein was extracted from the cecum tissues using RAPI lysis buffer, and quantified by the BCA assay. Protein samples were separated by sodium dodecyl sulfate-polyacrylamide gel electrophoresis (SDS-PAGE), and then transferred to PVDF membranes. After blocking with 5% skimmed milk for 1 h, the membranes were incubated overnight with TLR4 antibody (1:500, Wanleibio Co., Ltd.), Occludin antibody [1:1,000, Abcam (Hong Kong) Ltd.] and GAPDH [1:10,000, Abcam (Hong Kong) Ltd.] at 4°C. The membranes were washed with Tris-buffered saline containing 5% Tween-20 (TBST), and then incubated with HRP-conjugated secondary antibody at room temperature for 1 h. The protein bands were detected using an enhanced chemiluminescence (ECL) kit, and quantified by the gel imaging system (BIO-RAD, United States).

### Data Analysis

The experimental results were processed by GraphPad Prism five statistical software. All data are expressed as the mean ± standard error (SEM). Statistical differences between groups were determined by a multiple comparison ANOVA or a two-tailed paired *T* test, and *P* < 0.05 was considered statistically significant. The experiment used the software Elisacalic for calculations, and the data included the OD values of D-lactic acid and LPS.

## Results

### The Observation of the Stomach in the SD Rats at Different Time Points After MT

**FIGURE 1:**
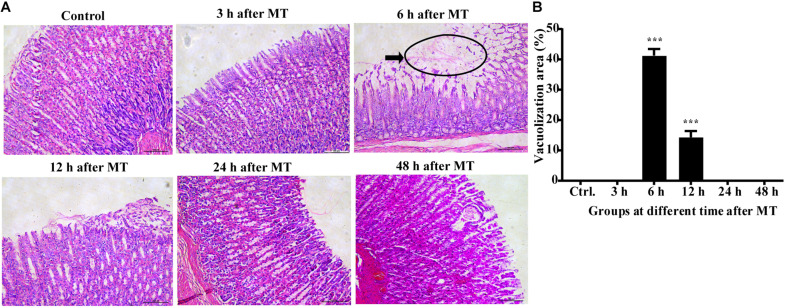
Changes in the gastric mucosa at different times after MT (200×). **(A)** HE staining of the stomach of the SD rats at different time points after MT. The black area shows obvious vacuolated changes at 6 and 12 h after MT. No obvious changes were observed in the control, 3, 24, and 48 h groups. **(B)** Mean (±SEM) percentages of morphologically vacuolized areas at 6 and 12 h (***P < 0.001 vs. the control).

The results of HE staining (Figure 1) of the stomach of the SD rats at different time points after MT revealed that vacuolization was rarely observed in the control group at 3, 24, and 48 h (0%), while there were few changes in the superficial gastric mucosal at 3 h. Homogeneous vacuolization was significantly increased to 41.27% at 6 h and 14.32% at 12 h compared with that of the control group (*P* < 0.001, *P* < 0.001). These results demonstrated that there was obvious damage in the gastric mucosa 6 and 12 h after MT and significant self-healing at 24 and 48 h after MT in the rats.

### Observation of the Ileum Mucosa of the SD Rats at Different Time Points After MT

**FIGURE 2:**
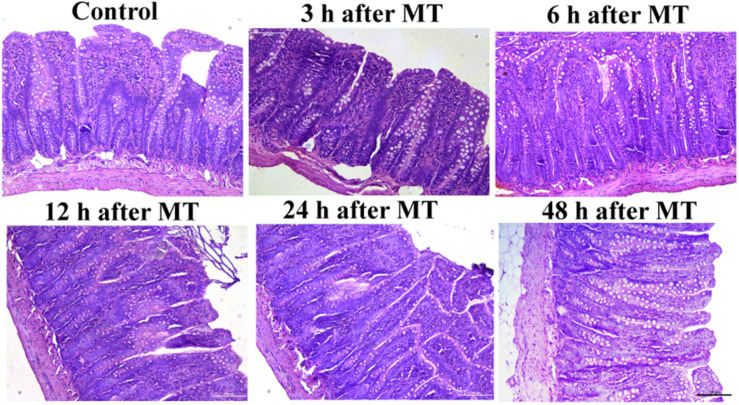
Changes in the ileum mucosa of the SD rats at different times after MT (200×). The morphology of ileum mucosa in all groups of different time points after MT were similar to that in the control group.

The results of ileal tissue staining showed no obvious injury to the mucosa at different time points after MT (Figure 2). There were no obvious injuries in the distal ileal mucosa of the SD rats after MT compared to those of the control rats. The brush-shaped edge was neat, and the goblet cells were active. The morphology was close to that of the control group. The morphology of the ileal mucosa in all groups at different time points after MT was similar to that in the control group.

### The Observation of the Cecal Mucosa in the SD Rats at Different Time Points After MT

**FIGURE 3:**
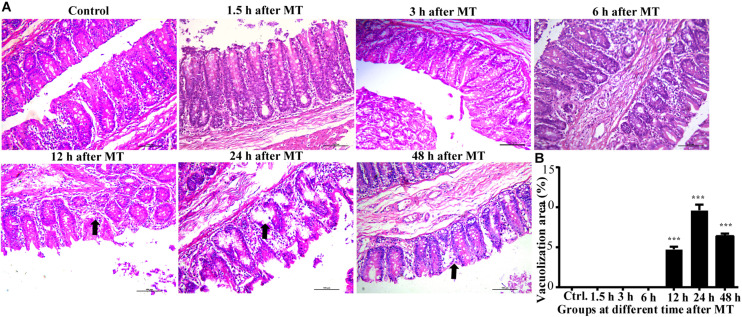
Changes in the cecal mucosa of the SD rats at different time points after MT (200×). **(A)** HE stainingof cecalmucosa in SD ratsat different time points after MT. The black arrow shows obvious vacuolated changes in 12, 24, and 48 h after MT. No obvious changes were observed in the control, 1.5, 3, and 6 h. **(B)** Mean (±SEM) percentages of morphologically vacuolizad area at 12, 24, and 48 h (***P < 0.001 vs. the control).

The results of HE staining showed that the injury in the cecum was more obvious than that of the stomach and the distal ileum after MT. This injury mainly manifests as the irregular shape of the brush edge of the mucous membrane, the disorder of the epithelial cells and contracted recesses. In addition, loosening of the structure was observed in some mucosal samples. These changes were observed at 12, 24, and 48 h after MT. These above features showed an increase in prominence. In this experiment, the injuries were most obvious at 24 h after MT, the area of the cecal mucosa vacuoles at 24 h was larger than that at 12 h after MT, and some vacuolated areas had missing patches (Figure 3).

### The Observation of the Cecal Mucosa in the SD Rats at Different Time Points After MT by an Oil Immersion Lens

**FIGURE 4:**
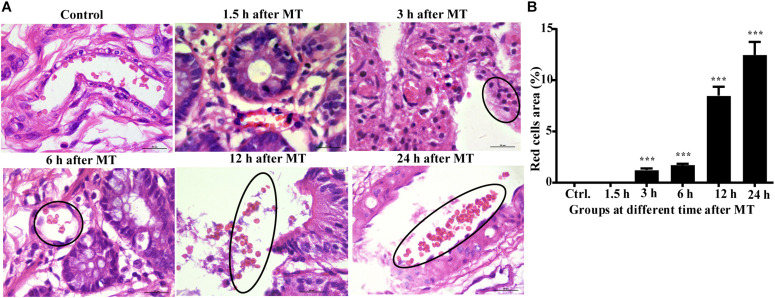
The morphology of the capillaries and red blood cells in the cecal mucosa of the SD rats at different time points after MT (1,000×). **(A)** HE staining in the cecal mucosa in SD rats at different time points after MT (1,000×). No obvious changes were observed in the control and 1.5 h. The areas in the black circle showed obvious red cells were found outside the capillaries in the time points of 3, 6, and 12 h after MT. There was obvious vascular wall damage at 24 h after MT. **(B)** Mean (±SEM) percentages of morphologically red blood areas at 3, 6, 12, and 24 h (***P < 0.001 vs. the control).

According to the above results, the cecum of the SD rats was obviously damaged after MT; therefore, the cecum may be the key location of the bacterial flora shift. If the bacteria enter the blood, the blood vessel wall may be damaged, and the blood cells may diffuse from the wall of the blood vessel. Therefore, we further examined the capillaries in the cecal mucosa. The results are shown in the black circle in Figure 4. The capillary wall of the control group was complete, and the red blood cells were observed in the blood vessel. In the trauma group, many red blood cells gathered and dispersed outside the vessel wall at 3, 6, and 12 h, and the blood vessel walls seemed to have been damaged in the group 24 h after MT (the black circle is shown; Figure 4).

### Changes in the Cecal Microvilli and Cell Connections in the SD Rats at Different Time Points After MT by Transmission Electron Microscopy

**FIGURE 5:**
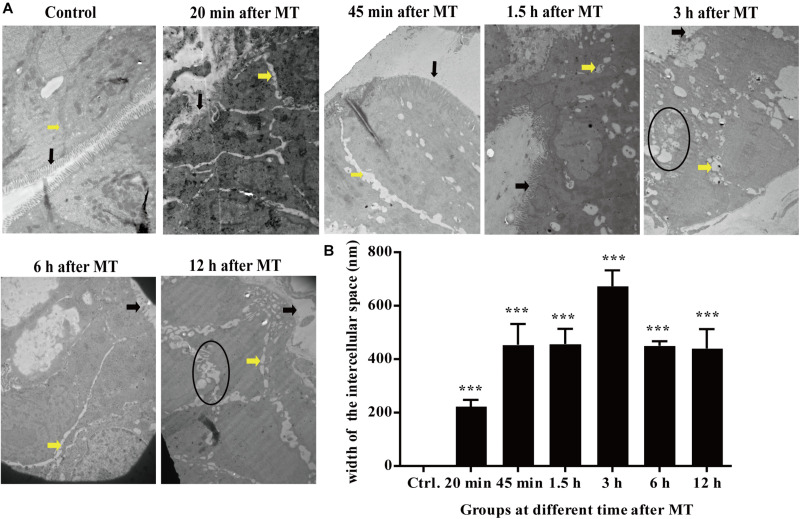
Changes in the microvilli and the cell junctions of cecal mucosa in the SD rats at different time points after MT (12,000×). **(A)** The black arrow in the figure was referred to the location of the microvilli. The yellow arrow was referred to the location of the cell junction. The areas in the black circle showed obvious vacuolated changes are found at 3 and 12 h. **(B)** Mean (±SEM) width of cell connections at different time points (***P < 0.001 vs. the control).

According to the Figure 5, the microvilli in the control group were neatly organized, and the cells were tightly connected without obvious intercellular space. The degree of injury to the microvilli and cell junctions at each time point after MT was higher than that in the control group. The cell connections were widened in each group after MT. The microvilli appeared sparse and scattered. With prolonged trauma times of 20, 45 min, 1.5, 3, 6, and 12 h, the cell junctions were further widened, and mucosal injury was generally aggravated. Some bacterial structures were observed in the widened intercellular space. The change in the tight junctions seemed to be smaller than that of the intermediate junctions and gap junctions, and there were vacuolar changes in some cells (Figure 5).

### The Observation of Bacterial Structures in the Intercellular Space After MT

**FIGURE 6:**
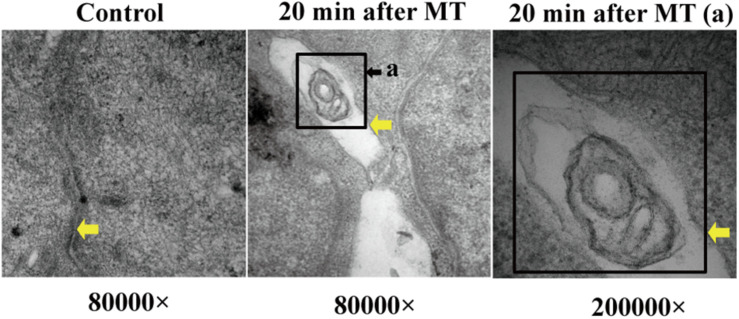
Bacterial structure between cell junctions. The position of the yellow arrows were defined as the cell junction or bacterial structure in the gap. The black square showed the bacterial structure. (a) Enlarged picture under the same vision at 20 min after MT (200,000×).

At 20 min after MT, transmission electron microscopy showed that the intercellular space of the SD rats increased significantly; at the same time, as shown in Figure 6, observations of some bacterial structure showed that the cecal mucosa of the SD rats was severely injured at 20 min after MT, indicating that a bacterial structure with a diameter of approximately 200 nm could freely enter along the widened cell junction channel. This finding further shows that the cell connections were destroyed.

**FIGURE 7:**
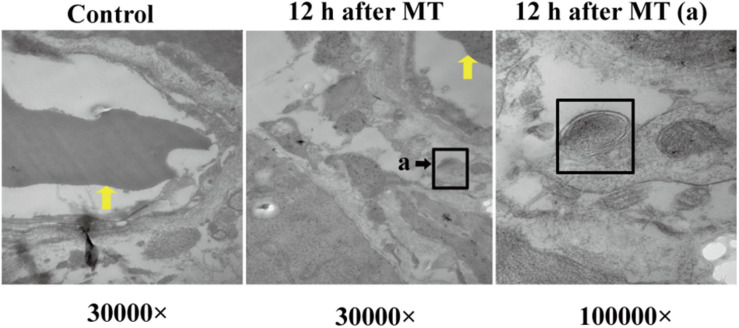
Bacterial structure near the capillary wall. The yellow arrow referred to the red cell. The black square referred to cross section structure of bacteria which has a double layer membrane structure. (a) Enlarged picture under the same vision at 12 h after MT (100,000×).

After MT, if bacteria translocate from the cecum to enter the blood circulation, they will appear near the capillaries of the cecal mucosal layer or even in the blood vessel wall. Therefore, we should further explore the capillary wall to identify bacteria or other pathogenic microorganisms. As shown in Figure 7, bacterial structures appeared outside the capillaries. They had double membrane structures, some of which seem to be moving into the capillaries or were located far from these structures.

In the transmission electron microscopy observations of the cecal mucosa of the SD rats after MT, the earliest time point with obvious damage was 20 min after MT. The cell connections were widened so that foreign bodies with a diameter of approximately 200 nm could enter. With prolonged time after MT, the intercellular space may become further widened, and bacterial structures appeared outside the capillary wall 12 h after MT.

### Changes in the Blood Markers Indicating Mucosal Injury

**FIGURE 8:**
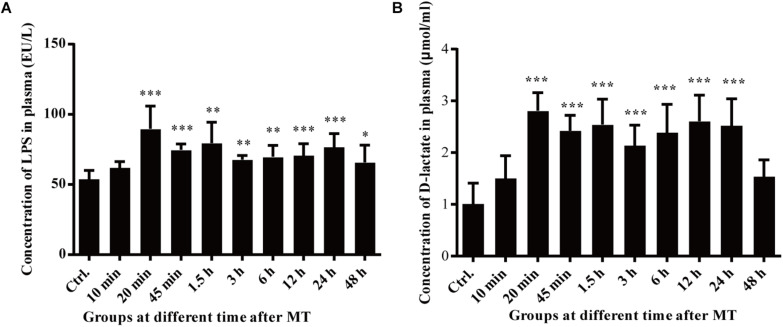
Changes in the plasma LPS and D-lactate concentrations of the SD rats at different time points after MT. **(A)** Changes in the plasma LPS concentration of the SD rats at different time points after MT (EU/L). There were statistically significant differences at 20, 45 min, 1.5, 3, 6, 24, and 48 h after MT (*P < 0.05, **P < 0.01 and ***P < 0.001 vs. the control). **(B)** Changes in the plasma of D-lactate concentration of the SD rats at different time points after MT (μmol/ml). There were statistically significant differences at 20, 45 min, 1.5, 3, 6, 12, and 24 h, after MT (*P < 0.05, **P < 0.01 and ***P < 0.001 vs. the control).

D-lactate and LPS were used as indirect markers for investigating the intestinal context in the experimental model. Changes in D-lactate and LPS in the blood at different time points after MT were further observed Figure 8. Compared with that in the control group, LPS in the plasma of the SD rats increased gradually after MT. At 20 min after MT, the concentration of LPS in the blood increased sharply and reached a peak. Compared with the 20-min LPS concentration group, the groups between 45 min and 24 h after MT showed a slight decrease in the plasma LPS concentration, which remained higher than that in the control group. The value of the group at 48 h after MT was significantly lower than that of the group at 24 h after MT (Figure 8A). As shown in Figure 8B, the concentration of D-lactate increased rapidly after MT. At 20 min after MT, the concentration reached a peak. Compared with the control group, there was a significant increase in the group at 20 min after MT. Compared with the D-lactate concentration of the group at a 24 h after MT, the group at 48 h after MT had a significant decrease. The plasma concentration of D-lactate remained high until 24 h after trauma. During this process, the concentration of D-lactate in the rat plasma was maintained at a high level from 20 min to 24 h.

### Morphological Imaging of Gastrointestinal Organs in the SD Rats at Different Time Points After MT

**FIGURE 9:**
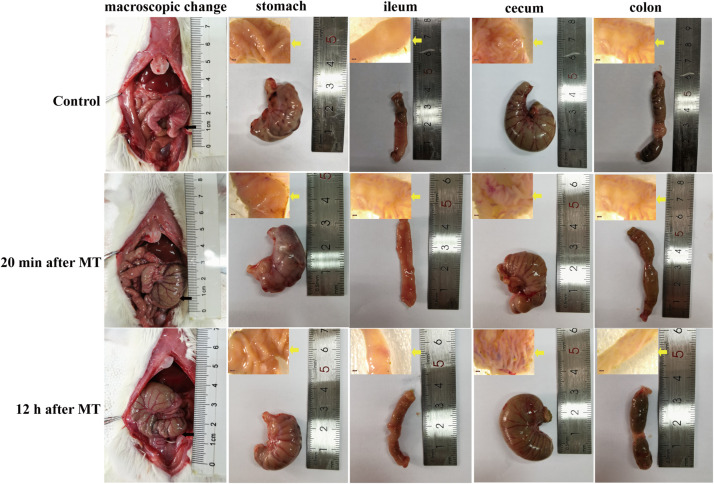
Macroscopic morphological images of gastrointestinal organs of the SD rats at 20 min and 12 h after MT. The black arrow in the figure was referred to the location of the cecum *in vivo*. The yellow arrow was referred to the anatomic mucosa of stomach, ileum, cecum and colon.

According to the results of HE staining above, we chose typical trauma times of 20 min and 12 h to observe the macroscopic image of SD rats after MT. As shown in Figure 9, slightly hyperemia was observed in the gastrointestinal tract organs of the SD rats is after MT compared with the control group, especially the cecum, which was also proved by the results of cecal mucosa dissection, while the macroscopic image changes of stomach, ileum and colon are not obvious.

### The Changes of Mucosal Permeability in Intestinal Mucosa

**FIGURE 10:**
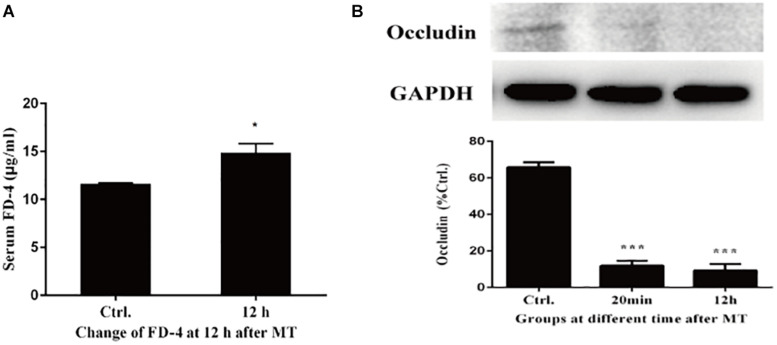
Changes of mucosal permeability in intestinal mucosa. **(A)** Serum concentration of FD-4 in SD rats at 12 h after MT (*P < 0.05 vs. the control). **(B)** The expression of Occludin at 20 min and 12 h after MT (***P < 0.001 vs. the control).

According to the results of transmission electron microscopy and blood markers, we further tested the mucosal permeability in intestinal mucosa by testing FD-4 and the expression of tight junction protein Occludin. The serum FD-4 concentration was increased at 12 h after MT compared with the control group (Figure 10A). As shown in Figure 10B, the expression of Occludin significantly decreased both at 20 min and 12 h after MT compared with the control group in cecum tissues. These data showed that the permeability cecal mucosa increased.

### Evaluating the Expression Level of Toll-Like Receptors 4 (TLR4)

**FIGURE 11:**
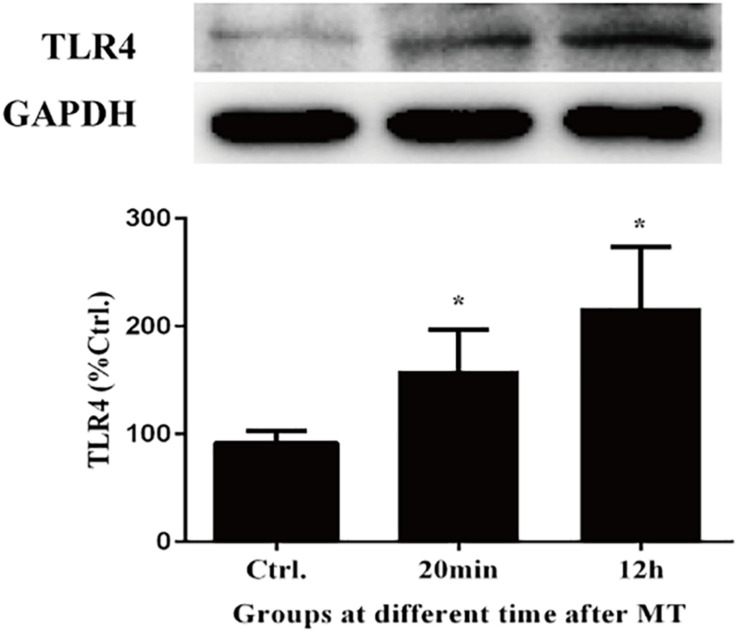
The changes of TLR4 in cecal tissue. There were statistically increased at 20 min and 12 h after MT (*P < 0.05 vs. the control).

We evaluated toll-like receptors 4 (TLR4) to validate the interaction of bacteria from the intestinal microbiota and gut. As shown in Figure 11, the expression of TLR4 increased both in cecal tissues at 20 min and 12 h after MT compared with the control group, which was accompanied with the increase of LPS.

## Discussion

Patients who experience MT and survive still face an increased risk of multiple organ failure (MOF), acute respiratory distress syndrome (ARDS), hospital-associated infection and sepsis, prolonged intensive care time and an increased risk of death (Lazarus et al., 2005; Glance et al., 2011; Gong et al., 2012; Cardozo Júnior and Silva, 2014; Ramsamy et al., 2017). The immune response caused by MT is closely related to the injury and symptoms of the patient (Atle et al., 2007). Since there is no open wound in the case of trauma, it is difficult and essential to determine the mechanism of bacterial translocation and identify treatments for the disease.

In research on mechanical trauma, it is necessary to first establish an animal model of mechanical trauma. At present, there are mainly two kinds of trauma models used in experiments: one is the local directional trauma model, which is mainly used for brain injury (Katz and Molina, 2018; Vennekens and Vankelecom, 2019), and the other is the non-directional trauma model (Noble and Collip, 1942; Tao et al., 2005), such as the systemic mechanical trauma model designed in this paper. This form of trauma triggers secondary injury of important organs of the body, such as cardiomyocyte apoptosis, which has been published in our previous research (Li et al., 2007a, b). There is no report about secondary injury to the digestive system caused by non-directional mechanical trauma. The aim of this study is as follows: (1) To observe gastrointestinal tract injury caused by non-directional mechanical trauma; (2) To explain the reason for the increase in TNF-α in the blood caused by mechanical trauma.

Previous studies of our research have shown that high levels of TNF-α released from the blood after mechanical trauma are a key factor that leads to cardiomyocyte apoptosis, and the peak value in the plasma is observed 1.5 h after mechanical trauma; at the same time, many studies have proven that TNF-α plays an important role in cardiac dysfunction, including endotoxemia, chronic heart failure, and myocardial obstruction (Hardaway, 2000; Hori and Kim, 2019; Sun and Yan, 2019). High levels of TNF-α are released from monocyte macrophages stimulated by LPS. LPS is a lipopolysaccharide on the cell wall of gram-negative bacteria, which becomes an endotoxin after bacterial death and lysis and causes the abovementioned inflammatory reactions. Because the body is suffering from mechanical trauma, that is, there are no open external wounds or external infection, LPS may be due to intestinal bacterial translocation. D-lactate and bacterial endotoxins are considered primarily markers of colon absorption, partially reflecting the permeability of the intestinal wall (Ruan et al., 2004; Wang et al., 2013); the cecum is the starting part of the colon. D-lactate and LPS are markers which can be seen in brain injury by mechanical trauma and may be related to other organs (Carpenter et al., 2015). Although there may be other tissues, D-lactate and LPS were used as indirect markers for investigating the intestinal context in the experimental model.

The gastrointestinal tract is the most important organ for microbial colonization in the human body (Josie and Young, 2019). The intestinal microflora concentration range is approximately 10^12^ to 10^15^ per gram of digested substance, from the upper part of the small intestine to the colon (Hazeldine et al., 2016). Under certain conditions, these bacteria can cause opportunistic infections and result in chronic inflammation and septic shock (Izcue et al., 2009; MacDonald et al., 2011; Lawrance, 2012; Fujimoto et al., 2013). Previous research shows that the normal intestinal flora will also produce disorders under various conditions such as dietary changes, abdominal surgery, radiation or antibacterial drugs (Van Vliet et al., 2010). The translocated gram-negative bacteria will initiate the excessive release of endotoxin, and the degradation products of other enteric gram-negative bacteria will increase significantly, which may be the main reason for the increase in LPS level (Wang et al., 2014). Injuries caused by MT may be similar to this process.

After MT, the SD rats had no obvious damage to the gastric and ileal mucosa, the cecal mucosa was obviously damaged, and the damage to the cecal mucosa was gradually aggravated over time after MT as was showed in the results above. At the same time, there was a sharp increase in the bacterial markers LPS and D-lactic acid in the plasma at 20 min after MT. The connections of the cecal epithelial cells were widened, the microvilli appeared sparse and showed shedding, and the crypts were deformed. Many red cells appeared outside the capillaries in the cecum of the SD rats 3 h after MT. Under a light microscope, injury in the cecal mucosa of the SD rats appeared to be more obvious after 12 than 48 h after MT. These injuries suggest that the cecal mucosa may be a key location of bacterial translocation after MT.

Although there are many intestinal bacteria and other microorganisms, due to the intestinal barrier, these microorganisms cannot invade the mucosa into the blood circulation. The mechanical barrier, an important component of the intercellular connection, includes the tight junctions. The tight junctions consist of blocking proteins, occludin proteins and cytoskeletal proteins. Previous studies have shown that hypoxia, inflammatory cytokines and bacterial antigens can affect the expression levels and assembly of the tight junction proteins, thereby affecting the function of tight junctions (Luo et al., 2012). The biological barrier is a symbiotic colony of intestinal flora on the surface of epithelial cells, and there is a relative balance between the epithelial surface of the intestine and the symbiotic flora, thereby maintaining homeostasis and preventing infection and inflammation (Geddes and Philpott, 2008; Maloy and Powrie, 2011). Therefore, we speculate that the mechanical barrier and the biological barrier of the rat cecum are significantly damaged by MT, which results in bacterial translocation.

To confirm this hypothesis, we performed further experiments. Changes in the cecal mucosa of the SD rats after MT were observed by transmission electron microscopy. With prolonged trauma times of 20, 45 min, 1.5, 3, 6, and 12 h, the cell junctions were further widened, and mucosal injury was generally aggravated (Figure 5). These mucosal injuries did not recover significantly with time after MT. Some bacterial structures began to appear in the widened intercellular space at 20 min (Figure 6). After MT, if bacteria translocate from the cecum to enter the blood circulation, they will appear near the capillaries of the cecal mucosal layer or even in the blood vessel wall. Therefore, we further examined the capillary wall to identify bacteria or other pathogenic microorganisms. As shown in Figure 7, bacterial structures appeared outside the capillaries at 12 h. Above all, non-directional trauma can cause widening of the cecal epithelial cell junctions in the SD rats, shedding of the microvilli, and destruction of the mechanical barrier at 20 min. At the same time, there was a sharp increase in the bacterial markers LPS and D-lactate acid in the plasma at 20 min after MT. As bacterial markers, LPS and D-lactate entered the blood, increased significantly after 20 min and maintained high levels 24 h after trauma (Figure 8). According to the results of transmission electron microscopy and blood markers, we further tested the mucosal permeability in intestinal cecal mucosa by testing FD-4 and the expression of Occludin at 12 h after MT and found that the intestinal mucosal permeability increased significantly (Figure 10). We detected TLR4 by Western Blot simultaneously and found that the expression increased significantly at 20 min and 12 h, especially at 12 h (Figure 11). TLR4 was activated in response to LPS stimulation, which suggests that the receptor was involved in mediating inflammatory responses. These results suggest that the cecal mucosa may be a key location of bacterial translocation after MT.

Based on this experimental study, non-directional mechanical trauma led to the following major changes in the intestinal tract: 20 min after MT, the tight junctions between the cecal epithelial cells of the SD rats began to be destroyed, and then, the connections between the intermediate junctions, gap junctions and other cells were significantly widened, and the mechanical barrier of the intestine was destroyed; furthermore, LPS and D-lactate, the products of gram-negative bacteria, increasingly moved from the intestine to the blood, causing the blood content to increase rapidly. According to previous studies, LPS binds to the TLR-4 receptor of monocyte macrophages, and the Toll-4 receptor is activated, which eventually leads to activation of the NF-KB molecule at the end of the signaling pathway and an increase in TNF-α, IL-1 and IL-6. These changes further aggravate the damage to the intestinal mucosa and increase the permeability of the mucosa (Ghareeb et al., 2016). The cecum is the primary site of gastrointestinal tract injury caused by MT. This experiment is important for early clinical intervention and prevention of secondary multiple organ injury and functional failure.

## Data Availability Statement

The original contributions presented in the study are included in the article/Supplementary Material, further inquiries can be directed to the corresponding author/s.

## Ethics Statement

The animal study was reviewed and approved by the animal studies committees of Dalian Medical University.

## Author Contributions

LL performed data analysis and wrote the manuscript. LW performed the experiments and data collection. CG performed helping the data analysis of transmission electron microscopy images in the experiment. HP performed some valuable opinions on process of experiment operation. HZ and DY participated in the construction of experimental model. LZ and SL were supervisors of the experiment and they performed in work on overall experiment design and manucsript writing. All authors contributed to the article and approved the submitted version.

## Conflict of Interest

The authors declare that the research was conducted in the absence of any commercial or financial relationships that could be construed as a potential conflict of interest.

## Supplementary Material

Supplementary Figure 1 Instrument used to perform the mechanical trauma.

Supplementary Figure 2 The control group.

Supplementary Figure 3 The MT group.

Supplementary Video 1 The control group.

Supplementary Video 2 The MT group.
