# Supplementary figures and images for: Effects of Non-directional Mechanical Trauma on Gastrointestinal Tract Injury in Rats
Source: Front Physiol. 2021 Apr 15;12:649554. doi: 10.3389/fphys.2021.649554 (PMC8081863; doi:10.3389/fphys.2021.649554)

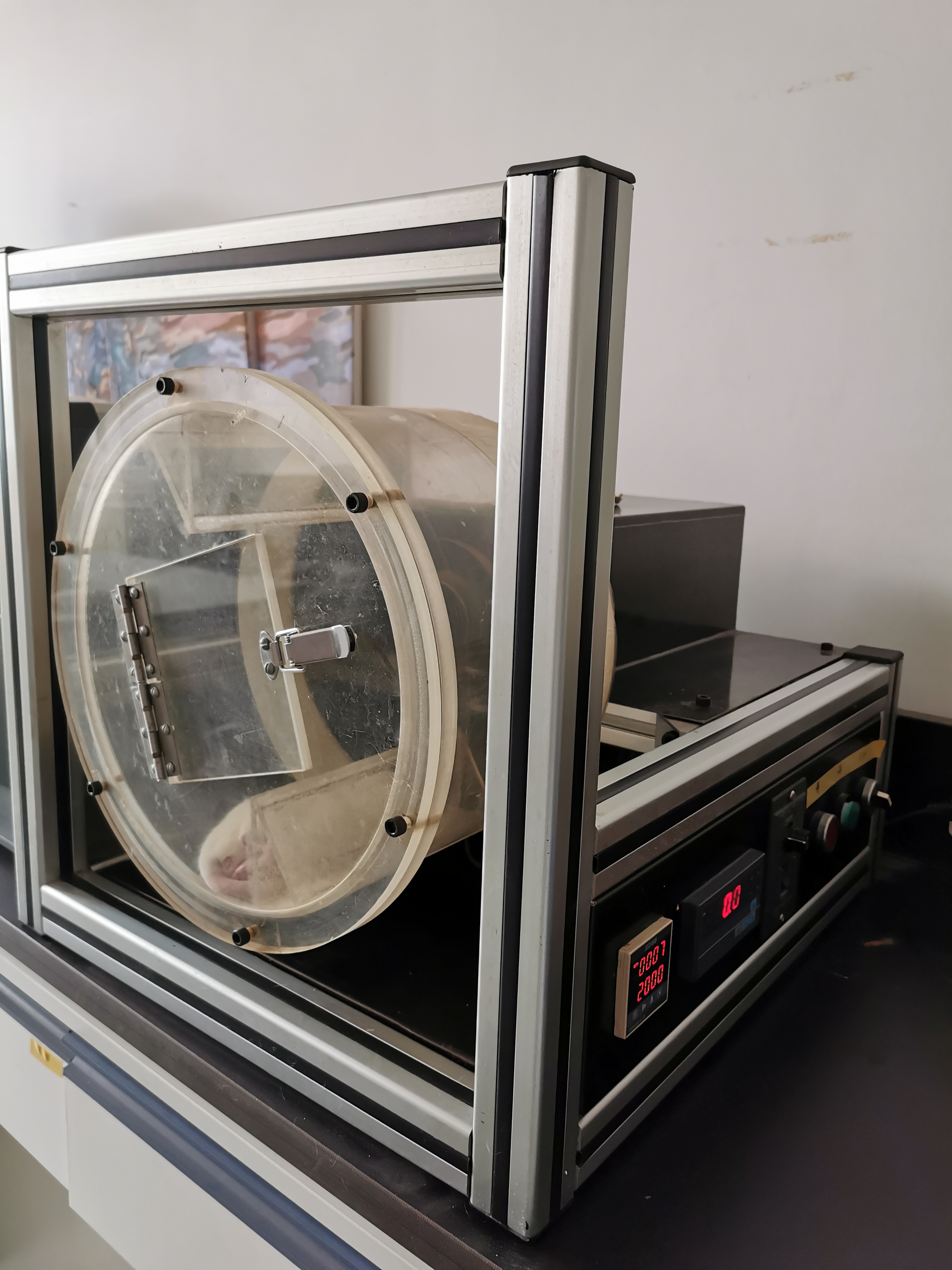

Supplement: Supplementary Figure 1 — Instrument used to perform the mechanical trauma. [file Image_1.JPEG]

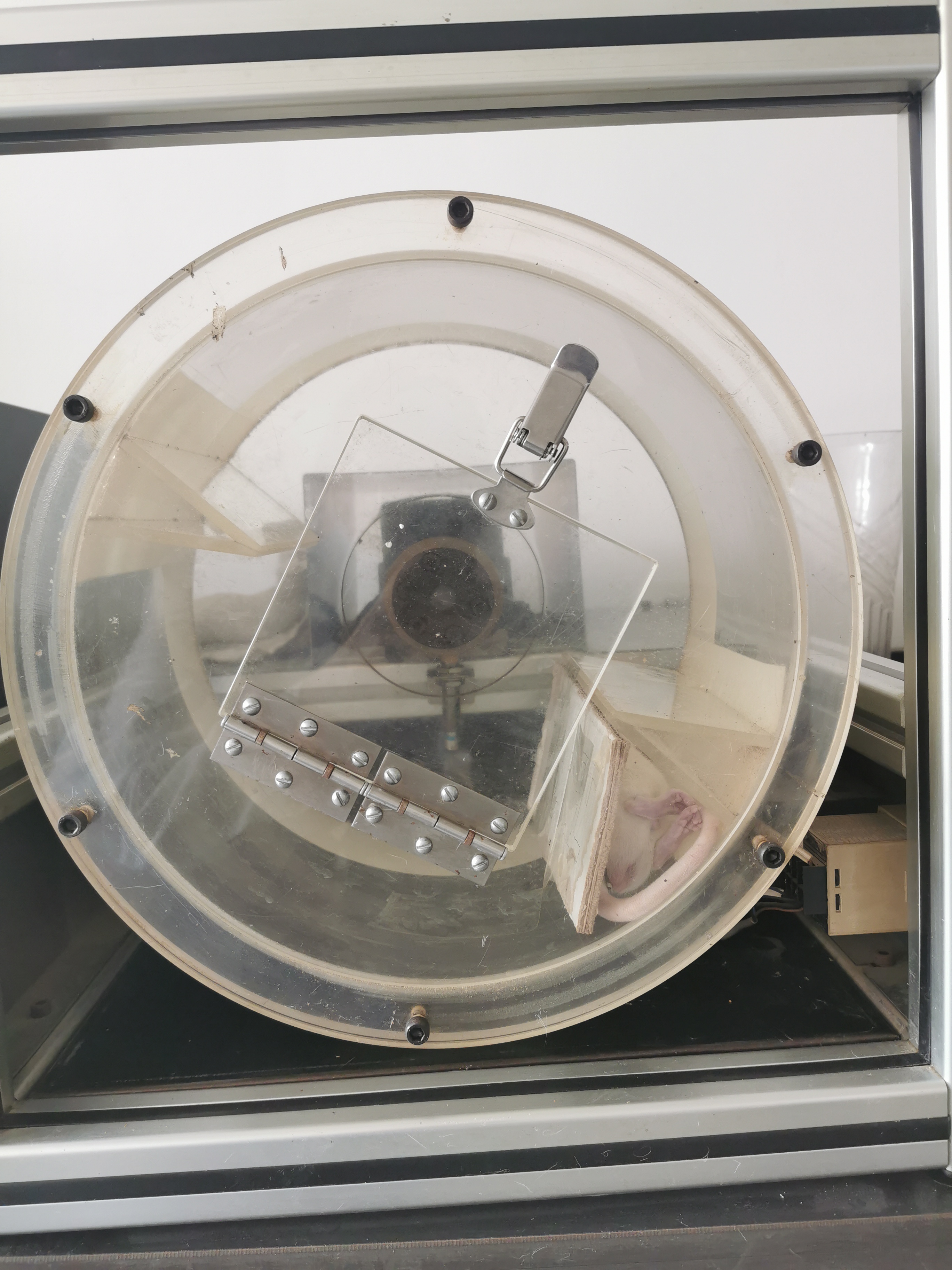

Supplement: Supplementary Figure 2 — The control group. [file Image_2.JPEG]

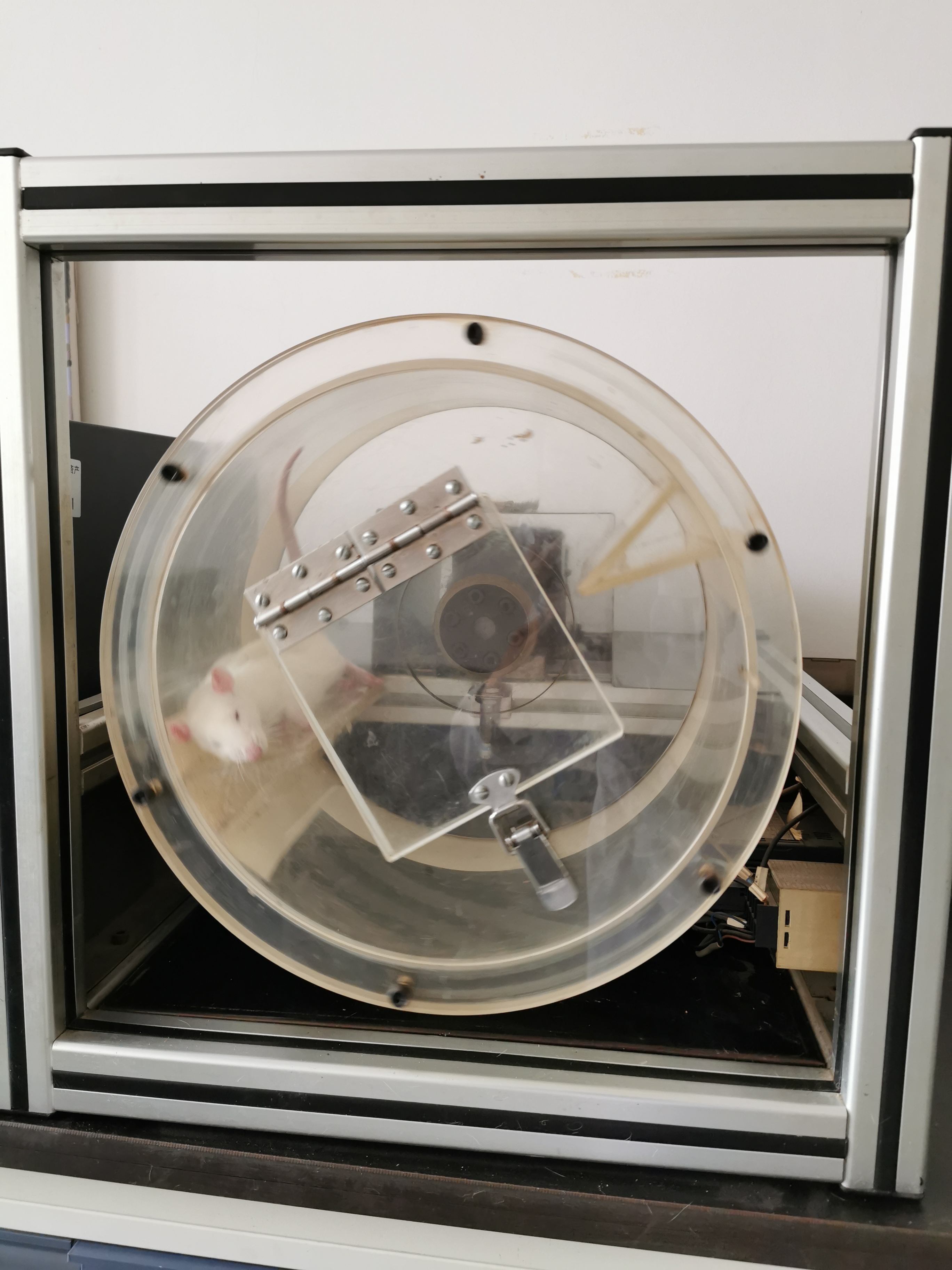

Supplement: Supplementary Figure 3 — The MT group. [file Image_3.JPEG]
